# Feasibility pilot of an adapted parenting program embedded within the Thai public health system

**DOI:** 10.1186/s12889-021-11081-4

**Published:** 2021-05-29

**Authors:** Amalee McCoy, Jamie M. Lachman, Catherine L. Ward, Sombat Tapanya, Tassawan Poomchaichote, Jane Kelly, Mavuto Mukaka, Phaik Yeong Cheah, Frances Gardner

**Affiliations:** 1 Centre for Evidence-Based Intervention, Department of Social Policy and Intervention, University of Oxford, Barnett House, 32 Wellington Square, Oxford, OX1 2ER UK; 2 Mahidol Oxford Tropical Medicine Research Unit, Faculty of Tropical Medicine, Mahidol University, Bangkok, Thailand; 3 MRC/CSO Social and Public Health Sciences Unit, University of Glasgow, Glasgow, UK; 4 Department of Psychology, University of Cape Town, Cape Town, South Africa; 5 Centre for Social Science Research, University of Cape Town, Cape Town, South Africa; 6 Centre for Tropical Medicine & Global Health, Nuffield Department of Medicine, University of Oxford, Oxford, UK

**Keywords:** Child abuse, Child maltreatment, Parenting, Positive parenting, Violence prevention, Abuse prevention, Thailand

## Abstract

**Background:**

This feasibility pilot of the Parenting for Lifelong Health for Young Children program in Thailand aimed to: 1) explore the feasibility of study evaluation approaches; 2) assess the feasibility of delivering an adapted program; 3) report indicative effects on child maltreatment and related outcomes; and 4) examine intervention content associated with key mechanisms of change perceived by caregivers and facilitators.

**Method:**

Sixty primary caregivers of children aged 2–9 years were recruited for an 8-week parenting program embedded within the local health system. Mixed-methods approaches included quantitative caregiver-report and observational data from standardized instruments, and qualitative data from individual and group interviews with caregivers and program facilitators. Analyses involved Wilcoxon signed-rank tests, paired t-tests, Friedman’s ANOVA, and thematic analysis.

**Results:**

Participants reported that most (65%) were grandparents or great-grandparents. Study retention and response rates were high, and enrolled caregivers attended an average of 93% of sessions. Primary outcomes showed caregiver-reported pre-post reductions in overall child maltreatment (*d* = − 0.58, *p* < 0.001), as well as reductions in physical (*d* = − 0.58, *p* < 0.001) and emotional abuse (*d* = − 0.40, *p* < 0.001). Combined caregiver report and observational assessments using the HOME Inventory showed reductions in abusive and harsh parenting (*d* = − 0.52, *p* < 0.001). Secondary outcomes demonstrated decreases in child neglect; dysfunctional parenting; poor child monitoring and supervision; parental sense of inefficacy; child behavior problems; daily report on child problem behavior; parent overall depression, anxiety, and stress; and attitudes supporting physical punishment and harsh discipline. There were increases in overall positive parenting, daily positive parenting behavior, as well as HOME Inventory assessments on parent-child relationships. Thematic analyses from interviews and focus group data identified six key program themes associated with strengthened parent-child relationships, reduced child behavior problems, improved attitudes and strategies toward discipline, and improved management of parental stress.

**Conclusions:**

This study represents one of few evaluations to test the feasibility of an evidence-based parenting program embedded within routine public health service delivery in a low- or middle-income country. Findings show preliminary effectiveness in reducing child maltreatment, improvements on 22 of 24 secondary outcomes, and perceived mechanisms of change that support quantitative findings. Prospects are promising for program scalability, pending randomized controlled trial results.

**Trial registration:**

11/01/2019, ClinicalTrials.gov, ID# NCT03539341.

**Supplementary Information:**

The online version contains supplementary material available at 10.1186/s12889-021-11081-4.

## Background

Violence against children (VAC) is a violation of child rights to protection under several international human rights treaties [1–4]. Estimates of the global prevalence of past year violence, including “moderate” forms such as spanking, is at least 50% – or exceeding 1 billion of the world’s children [5]. Estimates of such rates tend to be higher in low- and middle-income countries (LMICs) than in high-income countries [6], with minimum rates of past-year violence against children aged 2–14 years the highest in Asia, at 68% [5]. The most common perpetrators of VAC are household members [7], with this pervasive form of domestic violence similarly prevalent in Thailand. In a nationally representative household survey, adults reported that family members subjected 75% of children aged 1–14 years to at least one form of physical or emotional punishment in the past month [8]. The consequences of VAC are both immediate and long lasting, including delinquency, criminal activity, low educational performance, perpetration and victimization of intimate partner violence, and adverse mental health in childhood and adulthood [9–11].

Although harsh parenting and corporal punishment are often deemed normative in Asian LMICs [12], only a minority of adults in many LMICs believe such practices are necessary for childrearing [13] – suggesting they may be open to alternative forms of discipline. A well-established evidence base shows that social learning theory-based parenting programs can effectively reduce harsh parenting and prevent child maltreatment [14–17]. Such programs augment parenting skills by providing practical instruction on positive-parent child interactions, non-violent discipline techniques, socio-emotional coaching, problem solving, positive encouragement, and responsive supervision [18, 19], and may offer the non-violent methods that Asian primary caregivers want to learn [20, 21]. Interventions targeting difficult child behaviors and parental aggression are often similar to those which aim to prevent child maltreatment, and function by helping to disrupt coercive cycles of parent-child interactions [16, 18].

Several international development agencies are promoting an emphasis on the prevention of VAC and encouraging dissemination at scale [22]. However, such efforts face a number of challenges. First, many evidence-based parenting programs designed in high-income countries often charge expensive fees for licensing, materials, training, and support [23]. Second, while both transported and homegrown parenting programs can effectively reduce child maltreatment and harsh parenting in LMICs [16, 24, 25], some adaptation may be necessary [26]. Adapted interventions require empirical testing in new contexts, while mechanisms of change need to be examined in order to understand parent experiences and how this can inform efforts to maximize intervention effectiveness [27]. Finally, many LMICs must confront gaps in institutional ‘readiness,’ including whether there are sufficient human and technical resources, adequate institutional linkages and infrastructure, and sufficient funding and political will to assure scalability and sustainability [28].

In response, the Universities of Oxford, Cape Town, Stellenbosch, and Bangor, together with the WHO and UNICEF, developed Parenting for Lifelong Health for Young Children (PLH-YC), one of a suite of evidence-based, low-cost parenting programs freely available to LMICs [29]. PLH-YC is a group-based program that targets primary caregivers of children aged 2–9 years. It has shown reductions in child maltreatment and child behavior problems in randomized controlled trials (RCTs) in South Africa and the Philippines, along with improvements in positive parenting in the former and sustained child maltreatment effects at one-year follow-up in the latter^1^ [30]. Initially developed for low-income families in Cape Town, it is conducive to cultural and contextual adaptation in different LMIC settings [31, 32].

In Thailand, the University of Oxford, UNICEF, and the Thai Ministry of Public Health (MOPH) developed a partnership to adapt and test PLH-YC in accordance with the UK Medical Research Council guidelines on developing and evaluating complex interventions [33]. This iterative approach allows for the systematic adaptation of an intervention, designed to examine uncertainties, explore user and deliverer acceptability, and gauge outcomes through piloting prior to more stringent testing. Feasibility pilots may improve the quality of subsequent RCTs by focusing on the processes of the main study, such as recruitment, treatment, and assessments, while also exploring the suitability of outcome measures and the willing involvement of participants and deliverers [34]. In 2018, a formative evaluation informed study recruitment and adaptations to PLH-YC, taking into account potential barriers and opportunities for scaling up within routine public health services.^2^ During the current study, we conducted a single group pre-post feasibility pilot of the adapted PLH-YC program with low-income families in rural Thailand (*N* = 60) through the local public health system. Results from this pilot aim to inform further adaptation and testing in a pragmatic RCT using real-world conditions (*N* = 120) prior to wide-scale dissemination, if shown to be effective.

**Fig. 1:**
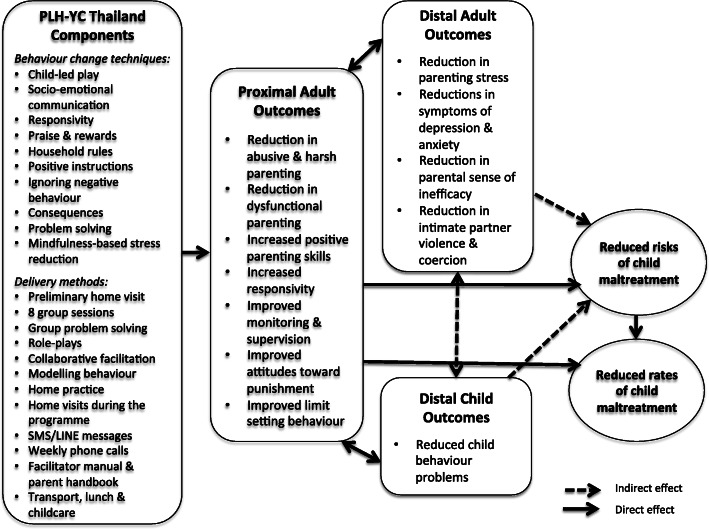
Theory of change model for PLH-YC Thailand

This study represents one of few evaluations to test the feasibility of an evidence-based parenting program embedded within routine public health service delivery in a LMIC. It is also the first known scientific study of such a program in Northeastern Thailand, a region that is home to nearly half (47%) of the country’s ‘skipped generation’ households – in which grandparents raise grandchildren in the absence of a mother and father [35]. The key research questions were: 1) Is a rigorous evaluation of a parenting program feasible within the public health system in terms of evaluation approaches, outcome measurement reliability, and adverse events monitoring?; 2) What is the feasibility of delivering an adapted version of PLH-YC to low-income primary caregivers with children aged 2–9 years, as measured by enrolment, attendance, completion, dropout, and fidelity?; 3) What are the indicative effects of the program on reducing VAC (primary outcomes) and associated risk factors (secondary outcomes) according to a hypothesized theory of change (see Fig. 1)?; and 4) What are the perceptions of caregivers and facilitators of program content associated with key mechanisms of change?

## Method

This mixed-methods feasibility study was pre-registered with ClinicalTrials.gov (NCT03539341). It is reported in accordance with the CONSORT 2010 extension to pilot and feasibility trials [36], as well as best practice guidelines on mixed methods research [37].

We embedded a qualitative component within a larger, quantitative study to provide an enriched understanding of the experimental pre-post outcomes, and to examine why the intervention may or may not have worked [38]. We collected qualitative and quantitative data in participants’ homes in parallel, with analysis for integration beginning after data collection had been completed [39], merging data by reporting results together and using each type to support or refute respective findings [37, 38]. Strategies for resolving discordance between findings included re-analyzing data, identifying possible theoretical explanations, and challenging construct validity [40]. Screening and pre-test data were collected during December 2018, 1 month prior to intervention delivery, with post-test quantitative and qualitative data collected approximately 1 month after program completion (late March to April 2019). Parent daily report assessments were conducted four times: at pre-test, by phone during the weeks following program sessions 2 and 6, and at post-test.

### Setting

The study was conducted in two districts of Udon Thani province in Northeast Thailand, the poorest and largest geographic region of the country [41]. Udon Thani has a population of almost 1 million people [42], with a high level of income inequality (Gini coefficient, 0.525) and a low rate of formal education (7.6 mean years of schooling) [43]. The majority of the population speaks the Isan language at home, although the Central Thai language is widely used in schools and government settings [44].

### Participants

As a feasibility pilot in preparation for an RCT, this study was not designed to reliably gauge significant intervention effects. Instead, the sample size was limited to 60 participants, which allowed for the formation of four parenting groups, each with 15 members. Sensitivity power analyses were conducted using a G*Power 3 calculator [45]. Input parameters included the use of two-tailed paired t-tests based on the study’s primary outcomes. Assuming a Type I error of *p* < 0.05, 80% power, and with no adjustments for attrition due to the intention-to-treat design, this sample size was sufficiently powered to detect a small significant intervention effect of *d* = 0.37 [46].

This study tested the feasibility of a recruitment strategy devised from formative evaluation findings and, given resource constraints, reflecting a pragmatic approach to service delivery. Caregiver participants were those with primary responsibility for the care of children aged 2–9 years. Village Health Volunteers (i.e., government community health workers) and teachers identified caregivers from low-income households with an annual income of approximately 100,000 Thai Baht (3310 USD) or less, who they thought would benefit from a program that would help them manage conflict with their child and difficult behaviors. Caregivers were then screened for supporting or engaging in violent discipline: researchers administered a 7-item screening instrument based on the Multiple Indicator Cluster Survey (MICS) child discipline module [8]. Participants passed the screening if they answered “yes” to one or more of six items regarding past month use of violent discipline, or responded “agree” or “strongly agree” regarding whether physical punishment was necessary for child rearing. Caregivers with multiple children aged 2–9 years were asked to select a target child with the most difficult behavior as the focus for the program, as these children are more at risk for violent punishment.

A trained research coordinator subsequently contacted referred caregivers by phone or home visit and invited them to participate in the study. The intervention was presented as a family support program in order to avoid potential stigma [47]. Eligibility criteria for participants included being aged 18 years or older and living in the same household as the target child for a minimum of four nights per week, so that there was sufficient time to apply learned parenting skills at home, as well as confirming that they were available and willing to attend group sessions on Sundays over the 8-week period.

### Adapted intervention

PLH-YC is a group-based, non-didactic, active learning-based parenting program, with content grounded in social learning principles and evidence-based principles and components from high-income countries [32]. The program is derived from the two-stage Hanf-model for parent management training, which focuses on strengthening positive parent-child relationships prior to engaging in behavior management and effective discipline strategies [48]. Core components include child-led play, child-directed speech, socio-emotional communication, praising and rewarding, instruction-giving, household rules and routines, and using ignore and consequences for non-compliance (see Fig. 1) [32]. The program is hypothesized (Fig. 1) to decrease risks and rates of child maltreatment by directly reducing abusive and dysfunctional parenting, as well as improving positive parenting skills, responsivity, monitoring and supervision, attitudes towards punishment, and limit setting behavior. These improved adult outcomes would then diminish parental mental health problems, parental sense of inefficacy, as well as intimate partner violence, while also reducing child behavior problems.

Various adaptations were made following the formative evaluation study in Thailand. The original 12-session version was reduced to eight sessions in order to better meet resource constraints (see Additional file 1). Cultural and contextual adaptations were also made by removing time-out content due to practitioner views that this technique was prone to misuse by Thai families, as discussed in more detail in the aforementioned formative evaluation paper. Other adaptations included modifications of language delivery, illustrated stories, scheduling, logistics, selection of facilitators, and scale-up strategies. In the adapted version, each session includes the following activities: (a) brief mindfulness exercise for stress management (i.e., “taking a pause”), (b) sharing of emotions to “check-in,” (c) physical exercise, (d) discussion on home practice activities from the previous session, (e) core lesson and discussions based on illustrated stories, (f) practicing new parenting skills through role-plays, (g) assignment of home practice activities based on the newly learned skills, (h) a closing mindfulness activity, and (i) sharing of emotions to “check-out.” English versions of the adapted PLH-YC facilitator manual and parent handbook are freely available online at: http://www.who.int/violence_injury_prevention/violence/child/PLH-manuals/en/index1.html.

### Delivery

During January to early March 2019, the adapted program was delivered in a mixture of Isan and Thai languages by paired facilitators to four caregiver groups. Each group met over eight sessions, with each session lasting 2–3.5 h. The facilitators conducted 1-h individual consultations at participants’ homes prior to the first session in order to introduce PLH-YC and establish tailored parenting goals. Home visits were also conducted during the course of the program to those caregivers who missed a session or needed additional support. Participants were provided with parent handbooks, which served as a resource for home practice, and were paired with a partner to foster peer support. Finally, facilitators made personalized weekly phone calls to each participant, and sent text message “boosters” to encourage skills practice at home.

### Measures

#### Study feasibility outcomes

In order to assess the suitability of study evaluation approaches, we examined: a) study recruitment and retention rates; b) outcome measure reliability; c) response rates of self-report outcomes and Home Observation for Measurement of the Environment (HOME) Inventory interview/observational assessments; and d) adverse events. *Recruitment rates* were determined at each point based on the number of potential participants who were contacted, met the inclusion criteria, passed the screening, and completed pre-test assessments following informed consent procedures. *Retention rates* were calculated based on the percentage of participants who completed pre-test assessments and then dropped out at any stage prior to completing post-test assessments. In addition, we assessed the reliability and response rates of outcome measures given that most had been validated in the United States and not in Thailand, with the exception of the Depression, Anxiety, and Stress Scale (DASS-21) [49]. Finally, in order to monitor adverse events, we maintained a log of child protection and adult welfare referrals, and utilized two-tailed tests of intervention effects to check for any signs of effects in the direction of harm.

#### Program feasibility aspects

The *enrollment rate* was based on the percentage of participants who attended at least one group session. The *attendance rate* was based on the number of enrolled participants who attended group sessions using facilitator-maintained records, while *dropout* was defined as the percentage of enrolled participants who missed three consecutive sessions and were not available for home visits. *Completion rates* were based on the percentage of enrolled participants who participated in at least 75% of the program. Finally, *fidelity*, or the extent to which the curriculum was delivered as planned [50], was assessed through weekly self-report fidelity checklists on session content completion. Fidelity scores were based on the ratio of activities implemented to the number of manualized activities, with a standard of 80% regarded as “high treatment fidelity” [51]. Additional aspects regarding program reach (e.g., obstacles to attendance, weekly home visit attendance, dosage of phone calls and text messages), program engagement, program delivery quality, and participant and facilitator acceptability are reported in a forthcoming process evaluation paper.

#### Demographic and socioeconomic measures

Household and family characteristics were measured by including items from the MICS Round 5 [8] (e.g., caregiver/child age, gender, education, marital status, relationship to target child; 24 items). Socioeconomic factors using items from MICS Round 5 and the 2015 Thai National Statistical Office Household Socioeconomic Survey [52] assessed relative poverty and living standards (e.g., income and benefits, household structure and assets, employment, food consumption, health care coverage; 16 items). An adapted version of the Medical Outcomes Study Short Form-12 Health Survey (3 items) [53] assessed caregiver physical health, while caregiver and child disability were measured using 2 items from the Washington Group [54]. Further, caregiver history of experiencing childhood abuse was assessed through an adapted version of the International Society for the Prevention for Child Abuse and Neglect (ISPCAN) Child Abuse Screening Tools Retrospective version (ICAST-R, 4 items) [55]. Past month food insecurity was measured using 5 items from the Hunger Scale Questionnaire [56].

#### Primary outcome measures

*Child maltreatment* (physical and emotional abuse, caregiver-report) was measured using the ISPCAN Trial Caregiver scale adapted for caregivers of children ages 2–9 (25 items, ICAST-TC) [57], which has been successfully used in a study of PLH-YC in the Philippines.^3^ The ICAST-TC measures incidence of child physical abuse (15 items) and emotional abuse (10 items) over the past month. Child maltreatment was also measured using an adapted version of the HOME Inventory acceptance subscale to examine *abusive and harsh parenting* (6 items; 2 via interview and 4 via observation during the assessment). The HOME Inventory was adapted by combining relevant items from the Early Childhood and Middle Childhood record forms [58].

#### Secondary outcome measures

Medical, physical, and educational *neglect* was assessed using the ICAST-TC [57]. *Positive parenting* was assessed using the Parenting Young Children Scale (PARYC), in which parents reported on the frequency of positive parenting, setting limits, and proactive parenting behaviors over the past month [59]. *Dysfunctional parenting* was measured using the over-reactivity sub-scale of the Parenting Scale [60]. *Parental depression, anxiety, and stress* were assessed using the DASS-21 [61]. *Poor child monitoring and supervision* were measured using the Alabama Parenting Questionnaire (APQ) poor monitoring/supervision subscale [62], while *child behavior problems* were assessed using the Eyberg Child Behavior Inventory (ECBI) [63]. *Caregiver-child relationships* were assessed through caregiver self-reports and observation of caregiver-child interactions using the adapted HOME Inventory [58], including parental responsivity and encouragement of child maturity sub-scales. *Parent sense of inefficacy* was assessed using the ICAST-TC inefficacy subscale (2 items), while *attitudes supporting physical punishment* were measured using the same item from the MICS 5 Child Discipline module administered during screening [64]. *Attitudes toward harsh discipline* were measured using the ICAST-TC attitudes subscale (4 items). *Intimate partner violence (IPV) and intimate partner coercion* were assessed using an adapted version of the Revised Conflict Tactics Scale Short Form (CTS2S) [65] and the WHO questionnaire on women’s health and life events [66], respectively. IPV and coercion were assessed given meta-analytic review findings that high family conflict is a strong risk factor for child physical abuse [67]. *Parent daily report on child problem behavior and positive parenting* was assessed using an adapted version of the Parent Daily Report checklist (PDR) [68]. For this assessment, caregivers reported by phone on whether a child problem behavior (e.g., lying, hitting; 34 items) or a parenting behavior and efficacy (e.g., yell shout, praise; 9 items) occurred in the previous 24 h. Feasibility of conducting PDR assessments on a monthly basis was examined, with time points two and three utilizing interviews by phone.

### Procedures

Questionnaires were translated into Thai and then back-translated into English, with a translation panel resolving translation discrepancies. Questionnaires were pre-tested with low-income caregivers of 2–9 year-old children in Udon Thani. Eleven Thai- and Isan-speaking research assistants used Computer-Assisted Self-Interviewing (CASI) methods with e-tablet technology to administer the questionnaires. To accommodate varying levels of literacy, research assistants read questionnaires aloud to participants. In addition, audio-CASI was used to administer sensitive items regarding child maltreatment and IPV. Past studies on sensitive issues have reported high acceptability of CASI in rural areas of Thailand [69], as well as in other LMICs [70].

Trained research assistants administered verbal and written informed consent, to account for varying levels of literacy. Monetary compensation was provided at each of seven data collection points, ranging from 50 to 150 Baht (1.65 to 4.95 USD). Refreshments, certificates, 150 Baht (4.95 USD) travel compensation per session, and transport (where needed) were provided as part of program delivery. Caregivers who participated in individual interviews received further compensation (100 Baht, 3.30 USD). Focus group discussion (FGD) participants received 200 Baht (6.60 USD), along with 150 Baht (4.95 USD) travel compensation. Caregivers were notified during the informed consent procedure that disclosures or observations of significant harm to children would be immediately reported. Except for these cases, or where participants requested referrals to local services, confidentiality was maintained. All referral cases continued to participate in the study.

Individual interviews with 11 caregivers were also conducted after the program. The sole male caregiver (caregiver #1) and a caregiver with moderate cognitive difficulties (caregiver #21) were purposively selected in order to include their unique perspectives, while high attending (*n* = 3) and low attending (*n* = 6) participants were randomly selected from those who attended either more than six sessions or fewer than seven sessions, respectively. One caregiver who did not attend any sessions was unavailable for interview despite repeated attempts.

Finally, a FGD was conducted with all eight facilitators, who included nurses, public health officers, a social worker, and a Village Health Volunteer employed within the public health system. Seven facilitators had university degrees, while one had a vocational degree. Prior to program delivery, facilitators had to successfully complete a five-day training.

### Quantitative data analysis

Response rates for each instrument were calculated at each time point. Outcome measure reliability and preliminary intervention effects were analyzed using SPSS 25.0. Cronbach’s alpha was used to assess internal consistency and reliability of each scale and subscale [71]. The distribution of each outcome variable was examined using Shapiro-Wilk tests. Wilcoxon signed-rank tests were conducted for non-normally distributed outcomes and paired t-tests for normally distributed data to compare pre-test and post-test scores [72]. Friedman’s ANOVA was conducted for parent daily report outcomes on child behavior and parenting, given its non-normal distribution and the administration of PDR assessments at four time points [72]. Cohen’s *d* effect sizes were calculated for all outcomes (small effects *d* = 0.20–0.49; medium effects *d* = 0.50–0.79; large effects *d* ≥ 0.80) [46, 73]. Analyses utilized an intention-to-treat approach, with all participants included regardless of program attendance or participation in all assessment points [74]. Multiple imputation was conducted in Stata 15.1 at the item level in order to account for missing data, with Little’s Missing Completely at Random (MCAR) test used with an expectation maximization algorithm in order to assess the randomness of missing data [75]. The Multiple Imputation by Chained Equations (MICE) method was used with a fully conditional specification and Markov Chain Monte Carlo (MCMC) algorithm with 10 maximum iterations. In order to support the conclusions based on imputed results, a complete case analysis was also conducted, including only those participants with complete data [76].

### Qualitative data collection and management

The first author, an English and Thai speaking woman with prior qualitative research experience, and a trained local researcher fluent in Thai and Isan languages, conducted interviews with participants and focus groups with facilitators. Interviews were conducted in participants’ homes and lasted 1–2 h, with parent handbooks as visual aids. Interviews were conducted in Thai, with Isan responses translated into Thai and English by the local researcher. The FGD with facilitators was conducted at a hotel meeting room 3 weeks post-intervention and lasted 3 h. Discussion was led by the first author. Interviews and the FGD were audio recorded, transcribed into Thai, and then translated into English, with written notes as backup.

Individual interviews and the FGD followed a standardized open-ended format with a structured guide approach, allowing flexibility to probe emergent themes and in-depth exploration of particular topics [77]. Broad questions examined the following themes: 1) perceived impact of the program on caregiver-child and family relationships; 2) engagement with and perceived relevance of parenting skills; 3) acceptability of delivery methods; and 4) acceptability of program scheduling, logistics, and materials. We report on findings from the first two themes with the latter two reported as part of the forthcoming process evaluation.

### Qualitative data analysis

A thematic approach within an experiential framework was used to analyze qualitative data [78]. Based on transcripts of two individual interviews and the FGD, an initial coding framework was developed by the first author as a lead coder and JK as an independent coder, using NVivo 12 Pro. On an iterative basis, the lead author merged each of the three sets of coded transcripts in succession, and then ran coding comparison queries to identify those codes with low inter-rater reliability (Cohen’s kappa < 0.7) [79, 80]. The coders then agreed on new emergent codes and clarified coding definitions, resulting in a final coding frame that consisted of three parent codes and 13 child codes. The lead coder then coded the remaining transcripts, and re-examined all coded data for patterned responses, broader meanings, and divergent viewpoints, selecting data extracts that represented key themes as well as areas of disagreement.

#### Trustworthiness

We employed several strategies to strengthen the trustworthiness – or quality and rigor – of our qualitative research [81]. First, we collected data from both caregivers and facilitators. Second, nine caregivers were randomly selected for interview in order to reduce selection bias. Third, to limit researcher bias, two persons independently coded three transcripts during the process of developing the coding frame. Finally, we maintained an audit trail to document methodological and analytical decisions, including records of discussions on coding relevance and application, and reflexive notes written by the first author [82].

### Merging of quantitative and qualitative data

We utilized the attendance registries to guide selection of caregivers for individual interviews, allowing for linkages at the study design stage. After conducting separate analyses of the quantitative and qualitative data in parallel, the qualitative coding was matched with the quantitative outcome data in order to maximize the strengths and minimize the weaknesses of each data type [39]. This allowed for a more thorough examination of the indicative effects of the intervention.

## Results

### Socio-demographic characteristics and risk factors for child abuse

Family characteristics and risk factors for child abuse at pre-test are summarized in Table 1. Adult participants were predominantly female (*n* = 59; 98.3%), married (*n* = 49; 81.7%), and grandparents or great-grandparents of children targeted in the program (*n* = 39; 65.0%), while the majority of others were biological parents (*n* = 20; 33.3%). The mean age was 47.2 years (SD = 15.4). Most had not completed secondary school (*n* = 51; 85.0%) and reported being able to read Thai easily (*n* = 36; 60.0%). Slightly fewer target children were female (*n* = 28; 46.7%), with a mean age of 4.9 years (SD = 2.0).

**Table 1:** Socio-demographic characteristics & child abuse risk factors among caregivers at pre-test

Sociodemographic characteristics and risk factors	Participants (***N*** = 60)
**Caregiver variables**	
Age, M, range (SD)	47.2, 18–68, (15.4)
Female, *n* (%)	59 (98.3)
Unemployed, *n* (%)	25 (41.7)
Secondary school not completed, *n* (%)	51 (85.0)
Married, *n* (%)	49 (81.7)
Relationship to target child	
Grandparent or great grandparent, *n* (%)	39 (65.0)
Biological parent, *n* (%)	20 (33.3)
Other relative, *n* (%)	1 (1.7)
Mainly speaks Isan language at home, *n* (%)	47 (78.3)
Can read easily, *n* (%)	36 (60.0)
Poor health in the past month, *n* (%)	11 (18.3)
Childhood experience of maltreatment, *n* (%)	44 (73.3)
Experiencing intimate partner violence, *n* (%)	24 (40.0)
**Child variables**	
Age, M, range (SD)	4.9, 2–9, (2.0)
Female gender, *n* (%)	28 (46.7)
**Household variables**	
Number of adults in the household, M, range (SD)	3.4, 1–5 (1.1)
Number of children in the household, M, range (SD)	1.9, 1–5, (0.8)
Neither biological parent living in the household, *n* (%)	16 (26.7)
Caregiver ran out of money to buy food ≥5 days in past 30 days, *n* (%)	7 (11.7)
Total monthly household income	
≤ 5000 Baht (160 USD), *n* (%)	8 (13.3)
5001–15,000 Baht (160–481 USD), *n* (%)	37 (61.7)
15,001–30,000 Baht (481–962 USD), *n* (%)	14 (23.3)
30,001–50,000 Baht (962–1603 USD), *n* (%)	1 (1.7)
House type	
Cement, brick or stone, *n* (%)	47 (78.3)
Wood, *n* (%)	8 (13.3)
Bamboo, plywood, or zinc (shack housing), *n* (%)	5 (8.4)

*M* mean

In terms of low socio-economic status and related risk factors for perpetration of child abuse, most reported a total monthly household income of 15,000 THB or less (≤ 495 USD; *n* = 45; 75%). Nearly one fifth reported experiencing poor health in the past month (*n* = 11; 18.3%), and 11.7% ran out of money to buy food for five or more days in the past month (*n* = 7). In over one quarter of households, neither biological parent of the target child lived in the home (*n* = 16; 26.7%). Rates of violence experienced by caregivers were high: 73.3% were victims of either physical or emotional maltreatment during childhood (*n* = 44), while 40.0% had experienced intimate partner violence in the past month at pre-test (*n* = 24).

### Study feasibility

**Fig. 2:**
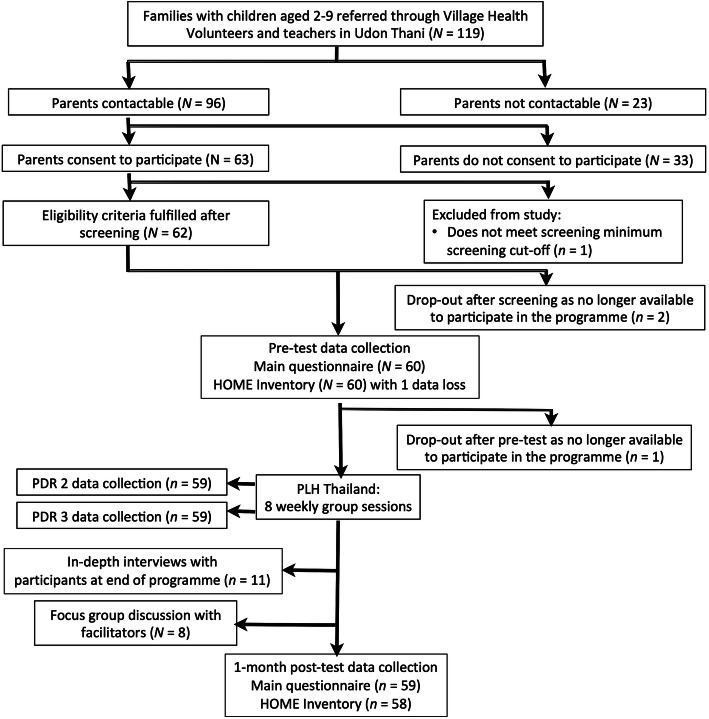
Study flow diagram

The flow diagram (Fig. 2) shows moderate-high levels of study recruitment, with 96 out of 119 referred caregivers (80.7%) successfully contacted, and 63 out of 96 contacted caregivers (65.6%) consenting to participate. A total of 52.9% of those referred thus consented to participate. Only four male referrals were provided, with three successfully contacted and one consenting to participate. The predominant reason given for declining consent was not having enough time to participate. A total of 62 out of 63 caregivers (98.4%) met eligibility criteria after screening, with 60 out of 62 caregivers (96.8%) ultimately participating in pre-test data collection. There was only one study dropout after pre-test, resulting in an overall retention rate of 98.3% at post-test. Study retention during HOME Inventory interview and observational assessments was high (100% at pre-test, 96.7% at post-test), as well as at PDR 2 and 3 time points (98.3%, both). Incomplete assessments or dropouts were due to the participant becoming unavailable to participate in PLH-YC, loss of contact with the participant, and one data loss from the HOME Inventory. An outlying case for child maltreatment outcomes at post-test assessment (caregiver #39) was identified using boxplots; subsequent contact with the participant revealed that responses entered into the tablet using the audio-CASI portion of the questionnaire for child maltreatment and intimate partner violence outcomes were incorrect and thus removed prior to analyses. Internal consistency of outcome instruments is reported in Additional file 2. Most alphas [71] (16 out of 27 values) were within acceptable ranges of 0.70 to > 0.90 at pre-test [83].

### Program feasibility

Rates of program enrolment, attendance, and completion were high. A total of 96.7% of caregivers recruited into the study attended at least one session, with enrolled caregivers attending 92.5% of all sessions. There was a 96.6% completion rate, with no program dropouts. Program adherence was also high, with facilitators implementing 95.5% of manualized activities based on self-reported fidelity checklists.

### Indicative program effects

#### Treatment of missing data

Little’s MCAR test on outcome variables found that the data was missing completely at random (χ^2=^8.987, df = 639, *p =* 1.000). Analysis showed that 76.0% of outcome variables had at least one instance of missing data, primarily due to dropout of one participant following pre-test, with a total of 1.16% missing values. Convergence was not achieved on 25 out of 487 variables, affecting 10 scales. A modified intention-to-treat approach was thus subsequently undertaken, utilizing all available imputed data in the computation of average scores for all scales (Tables 2 and 3) [84], while the complete case analyses are available in Additional file 3. Sensitivity analyses showed no significant differences between the complete cases and imputed datasets. This lack of differences was expected due to the very high study retention rates, as an intention-to-treat approach offers less advantage as a protection against bias compared to situations of high study attrition. However, as a matter of statistical principle, it is important that intention-to-treat results are presented to conform to accepted standards of reporting study findings.

**Table 2:** Outcomes at pre- and post-test for pooled imputed datasets

Outcome	Pre-test ***M*** (***SD***)	Post-test M (***SD***)	Test statistic* (***p***)	Effect size^**b**^	***N***
**Primary outcome**					
Child maltreatment - physical & emotional abuse (ICAST-T), /200^ac^	7.68 (7.02)	3.63 (3.81)	**- 5.00 (< 0.001)**	**- 0.58**	59
Physical abuse subscale, /120^c^	3.58 (3.92)	1.31 (2.01)	**- 4.58 (< 0.001)**	**- 0.58**	59
Emotional abuse subscale, /80^c^	4.10 (4.43)	2.32 (2.90)	**- 4.19 (< 0.001)**	**- 0.40**	59
HOME Inventory: Abusive & harsh parenting, /6	1.13 (1.05)	0.58 (1.01)	**- 3.71 (< 0.001)**	**- 0.52**	60
**Secondary outcomes**					
Overall positive parenting (PARYC), /126	69.00 (17.14)	80.75 (16.27)	**4.99**^**d**^ **(< 0.001)**	**0.69**	60
Supporting positive behavior subscale, /42	24.83 (5.60)	28.73 (5.14)	**5.00**^**d**^ **(< 0.001)**	**0.70**	60
Setting limits subscale, /42	21.90 (7.89)	26.95 (6.13)	**4.91**^**d**^ **(< 0.001)**	**0.64**	60
Proactive parenting subscale, /42	22.27 (6.93)	25.07 (7.28)	**2.62**^**d**^ **(0.011)**	**0.40**	60
Dysfunctional parenting (PS), /70	26.13 (9.06)	19.90 (8.13)	**- 5.14 (< 0.001)**	**- 0.69**	60
Poor child monitoring & supervision (APQ), /55	15.87 (4.94)	14.43 (4.14)	**- 2.67 (0.008)**	**- 0.29**	60
Neglect (ICAST-T), /48^c^	1.33 (2.22)	0.34 (2.30)	**- 3.35 (0.001)**	**- 0.45**	59
Overall depression, anxiety, and stress (DASS-21), /126	7.75 (7.26)	4.27 (4.83)	**- 3.44 (0.001)**	**- 0.48**	60
Depression subscale, /42	4.70 (5.80)	2.17 (3.54)	**- 3.31 (0.001)**	**- 0.44**	60
Anxiety subscale, /42	4.63 (4.95)	2.43 (3.15)	**- 3.11 (0.002)**	**- 0.45**	60
Stress subscale, /42	6.17 (5.07)	3.93 (4.33)	**- 2.74 (0.006)**	**- 0.44**	60
Attitudes supporting physical punishment (MICS), /23	1.98 (1.23)	0.93 (1.02)	**- 5.13 (< 0.001)**	**- 0.86**	60
Attitudes toward harsh discipline (ICAST-T), /20	10.30 (2.01)	8.47 (1.72)	**- 5.13 (< 0.001)**	**- 0.91**	60
Child behavior problems (ECBI) – Intensity subscale, /252	100.98 (28.66)	78.28 (27.38)	**- 5.28 (< 0.001)**	**- 0.79**	60
Child behavior problems (ECBI) – Problems subscale, /36 ^c^	7.68 (9.34)	1.77 (4.83)	**- 5.28 (< 0.001)**	**- 0.63**	60
Parent sense of inefficacy subscale (ICAST-T), /16	3.82 (3.42)	1.92 (2.42)	**- 4.00 (< 0.001)**	**- 0.56**	59
HOME Inventory: Overall caregiver-child relationships, /27	21.17 (3.63)	24.08 (2.66)	**5.36 (< 0.001)**	**0.80**	60
Parental responsivity subscale, /16^c^	13.17 (2.61)	14.25 (1.91)	**3.30 (0.001)**	**0.42**	60
Encouragement of child maturity subscale, /6^c^	4.50 (1.42)	5.58 (0.91)	**4.93 (< 0.001)**	**0.76**	60
Intimate partner violence subscale (CTS2S)/48 ^c^	1.08 (1.60)	0.54 (1.05)	**- 2.06 (0.039)**	**- 0.34**	48^e^
Intimate partner negotiation subscale (CTS2S)/16	2.81 (2.92)	3.15 (4.17)	- 0.32 (0.975)		48^e^
Intimate partner coercion (WHO), /80 ^c^	3.50 (5.12)	2.02 (3.50)	- 1.51 (0.132)		48^e^

Statistically significant differences (*p* < 0.05) between pre- and post-test are in bold

*Unless otherwise noted, standardized test statistics are from Wilcoxon Signed Rank tests

^a^Value indicates the maximum possible total score

^b^Cohen’s *d*

^c^Complete imputations not possible due to lack of convergence

^d^Test statistic from paired samples t-test

^e^Wilcoxon Signed Rank test calculated for 43 participants who were in a relationship at pre- and post-test

**Table 3:** PDR assessment outcomes and comparisons at four time points for pooled imputed datasets

Outcome	Pre-test ***M*** (***SD***)	PDR#2 ***M*** (SD)	PDR #3 ***M*** (***SD***)	Post-test ***M*** (***SD***)	Test statistic* (***p***)	***N***	Comparison	Test statistic* (***p***)	***SE***	Effect size^**b**^
Parent daily report (PDR) on child problem behavior, /34^ac^	6.87 (5.56)	6.72 (5.24)	4.73 (4.42)	3.70 (3.86)	**35.22 (< 0.001)**	60	Pre-test, PDR#2	- 0.02 (0.944)	0.24	
							PDR#2, PDR#3	**- 0.83 (< 0.001)**	0.24	**- 0.38**
							PDR#3, Post-test	- 0.23 (0.322)	0.24	
							Pre-test, Post-test	**- 1.04 (< 0.001)**	0.24	**- 0.57**
Parent daily report (PDR) on positive parenting behavior, /9	7.17 (1.42)	7.97 (1.09)	8.27 (0.97)	8.32 (1.00)	**45.98 (< 0.001)**	60	Pre-test, PDR#2	**0.76 (0.001)**	0.24	**0.57**
							PDR#2, PDR#3	0.37 (0.120)	0.24	
							PDR#3, Post-test	0.06 (0.805)	0.24	
							Pre-test, Post-test	**1.18 (< 0.001)**	0.24	**0.81**

Statistically significant differences (*p* < 0.05) between comparisons are in bold

*Test statistics are from Friedman’s ANOVA

^a^Value indicates the maximum possible total score

^b^Cohen’s *d*

^c^Complete imputations not possible due to lack of convergence

#### Primary outcomes

Tables 2 and 3 summarize means, standard deviations, test statistics, and *p*-values for all outcome variables using multiple imputation analyses at pre- and post-test, as well as for PDR assessments at four time points. Analyses showed a medium size effect (*d* = − 0.58) on child maltreatment (physical and emotional abuse), with a significant reduction following the intervention (*p* < 0.001) from a mean score of 7.68 (SD 7.02) to 3.63 (SD 3.81). Prior to PLH-YC participation, 90% of caregivers (*n* = 54) reported perpetrating at least one instance of physical or emotional abuse against the target child in the past month, falling to 71.2% post-intervention. Significant reductions (*p* < 0.001) on mean scores of child physical abuse represented a medium size effect (*d* = − 0.58), from 3.58 (SD 3.92) to 1.31 (SD 2.01). There was a significant yet small size effect (*d* = − 0.40) on child emotional abuse (*p* < 0.001), decreasing from a mean score of 4.10 (SD 4.43) to 2.32 (SD 2.90). Combined observation and interview assessments using the HOME Inventory found a medium size effect (*d* = − 0.52) on abusive and harsh parenting, with a significant post-intervention reduction (*p* < 0.001) from a mean score of 1.13 (SD 1.05) to 0.58 (SD 1.01).

#### Secondary outcomes

Child neglect was significantly reduced following the intervention (*d* = *−* 0.45; *p =* 0.001). Caregivers also reported decreases in dysfunctional parenting *(d* = − 0.69*; p* < 0.001), poor child monitoring and supervision (*d* = − 0.29; *p* = 0.008), and parental sense of inefficacy (*d* = − 0.56; *p* < 0.001). Child behavior problems were also significantly reduced, on both the intensity subscale (*d = − 0,79*; *p* < 0.001) and problem subscale (*d* = − 0.63; *p* < 0.001). There were significant reductions in caregiver overall depression, anxiety, and stress (*d* = − 0.48; *p* = 0.001), as well as across all subscales. Average scores of caregiver attitudes also significantly decreased, including attitudes supporting physical punishment (*d =* − 0.86; *p* < 0.001), and attitudes toward harsh discipline (*d = −* 0.91; *p* < 0.001).

There were significant increases in overall positive parenting (*d =* 0.69; *p* < 0.001), as well as for the three sub-scales on supporting positive behavior (*d* = 0.70; *p* < 0.001), setting limits (*d* = 0.64; *p* < 0.001), and proactive parenting (*d =* 0.40; *p* = 0.011). HOME Inventory assessments found significant improvements in overall caregiver-child relationships (*d =* 0.80; *p* < 0.001), as well as on the parental responsivity subscale (*d* = 0.42; *p* = 0.001) and encouragement of child maturity subscale (*d* = 0.76; *p* < 0.001).

In addition, there was a significant decrease in parent daily reports on child behavior problems from pre- to post-test (*d* = − 0.57; *p* < 0.001). The parent daily report on positive parenting behavior also significantly increased (*d =* 0.81; *p* < 0.001).

Finally, there was a significant reduction in intimate partner violence (*d* = − 0.34; *p* = 0.039). However, this was not significant for reports of intimate partner coercion (*p* = 0.132), with a large standard deviation indicating a wide variation in responses. There was also no significant improvement in intimate partner negotiation (*p =* 0.975).

### Perceived key mechanisms of change

**Fig. 3:**
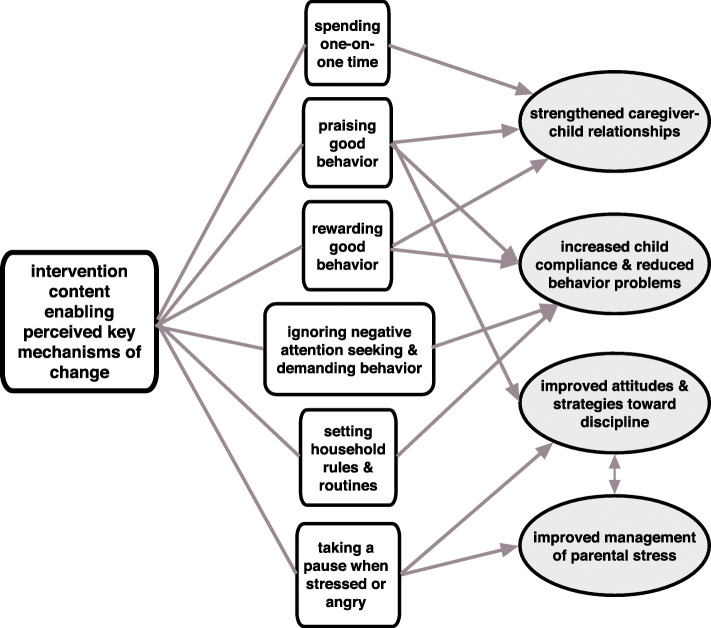
Conceptual model of intervention content enabling perceived key mechanisms of change

Views expressed during caregiver interviews and the FGD with facilitators indicated that six program themes contributed to four key changes in caregiver-child relationships and interactions. Figure 3 depicts the conceptual model of the interaction between these themes, as part of an overall model of perceived key mechanisms of change.

#### Strengthened caregiver-child relationships

Many caregivers expressed that their relationships with their children had strengthened due to program participation, and associated the program themes of one-on-one time, as well as praising and rewarding good behaviors, as key factors. A grandparent of a 4-year-old girl remarked, “I never got to play with my grandchildren like this [before]. Ever since I went to the training, I spent time playing with them …. It [their relationship] is a lot better than at the start of the workshop” (caregiver #16, interview). Facilitators noted that many caregivers shared that they liked the one-on-one time technique the most. One facilitator stated, “They can feel that when they spend time with their child, it really is good. The relationships became closer and improved” (facilitator #5, FGD). Two caregivers, however, struggled with allowing the child to lead or with spending focused time with the child during play, which suggests that the time spent together may be more important than the specific nature of how that time is utilized. According to a mother of a 5 year-old boy, “He cannot lead …. I have to be the leader for him and lead him to play” (caregiver #58, interview).

Caregivers also widely referred to the use of positive reinforcement for good behavior. Some associated praising with bonding with their children, as shared by one grandparent of an 8-year-old boy: “If I praised him more often, I feel that our relationship as grandmother and grandson – we love each other more” (caregiver #4, interview). Rewarding positive child behavior through expressions of physical affection, such as hugs and *horm gaem* (sniffing cheeks as a form of a kiss), were also mentioned. A mother of a 4-year-old girl described how she used such displays of affection as a reward 4–5 times per week, which resulted in reciprocation and a closer relationship: “Now my child likes to hug me, but before I never really did it. I went to the workshop and I came home to hug my child – now my child hugs [me] and sniffs [my cheeks] all the time” (caregiver #45, interview).

#### Increased child compliance and reduced behavior problems

Most caregivers and facilitators also indicated that their children were more compliant and exhibited fewer behavior problems after the program, mirroring quantitative data trends. Caregivers associated this decline with four program themes: praising, rewarding, ignoring negative attention seeking and demanding behavior, and setting household rules and routines. One mother of a 3-year-old boy expressed how praise effectively reinforced positive child behavior following an instruction: “I praised him every time …. I asked him to turn on the light … he did it and I said, ‘You are very good.’ He liked it and he was happy …. [Now] he wants me and everybody in the house to praise him” (caregiver #30, interview). Caregivers also highlighted that ignoring negative behaviors, especially demands for toys or snacks in stores, was effective in reducing their occurrence. One grandparent of an 8-year-old boy stated, “I think it [ignoring] is good, the best one that I can do … when he wants a toy and sometimes we couldn’t afford it … I would walk away …. When I came back he would be better and forgot about it” (#1 caregiver, interview). In addition, caregivers mentioned that household rules were successful in reinforcing compliance, with such rules allowing for the clear articulation of expected behavior. A great grandparent of a 4-year-old girl explained why a curfew she instituted during the course of the program was effective: “She listens well and I don’t really have to repeat myself. If I said you have to be home by … 6:00 [p.m.], then she has to come home like I said. I’d say it has changed for the better” (caregiver #20, interview).

#### Improved attitudes and strategies toward discipline

Parents expressed that they had altered their views on the appropriateness of corporal punishment, indicating that ‘taking a pause’ – the mindfulness-based stress reduction technique – helped bring about this change. One grandmother of a 3-year-old boy noted:

When [child] was fussy and wanted to eat this or that, if I got angry, I would stop first. I’m afraid that if I get angry, I would hit him … I would go outside for a break …. Then I would be cooled down …. [I use ‘taking a pause’] often, 2-3 times per week. (#51 caregiver, interview)

Caregivers also highlighted that using praise, which helped to build positive relationships with their children, decreased the necessity of using violent discipline. One grandparent of a 5-year-old boy who aimed to stop his use of cursing explained: “He used to say ‘*ay-hah*, *ay-kwai*’ [swear words for ‘damn’ and ‘buffalo’ or an ‘idiot’]. I would’ve just slapped his mouth! ...I kept praising him until he started to change …. ‘Oh, [child]! How are you so good?’” (#21 caregiver, interview). A grandparent of an 8-year-old boy, who used to frequently hit him, noted: “Praising is good because it’s better than using violence with him – using violence is not right, to put it simply” (#1 caregiver, interview). However, this attitude did not translate to a reduction in emotional abuse for a few parents. A grandparent said that she had “changed from hitting” to threatening her 3-year-old grandson that if he returned home late, “I will run away … if you come home and you don’t see me, [you] will live alone” (#51 caregiver, interview).

#### Improved management of parental stress

Many caregivers also described their use of ‘taking a pause’ to manage parental stress and to self-regulate their emotions, which was also linked to the avoidance of violent discipline. One mother of a 3-year-old boy shared, “I use this [‘taking a pause’] most often …. When my child has a tantrum, I remain calm …. I fully control my emotions …. I [used to] scold him. Now, I am much calmer. Before, I was about to kill him” (#30 caregiver, interview). Another caregiver, a grandmother of a 4-year-old girl, explained:

I used it [‘taking a pause’] when I’m upset …. The grandchildren would annoy me, so I used it …. I didn’t know how to do it before and I used to cry out loud. Then I remembered … to calm down, and that it works. This [technique] is my favorite. (#16 caregiver, interview)

Further, caregivers described the utility of using this skill with their children and other adults in the household, noting that it had also strengthened these relationships. A mother of a 3-year-old girl explained, “I use it [‘taking a pause’], my mother uses it, my father uses it, [and] my grandfather uses it because he loves his granddaughter so much …. After the training, it was effective in many ways” (#40 caregiver, interview).

## Discussion

This feasibility study is one of few evaluations of parenting programs in Thailand and the wider East and Southeast Asian region that aim to reduce VAC [25]. It is also one of few studies that included grandparents as the majority of participants and tested the feasibility and indicative effects of a parenting intervention embedded within the public health system. The potential for program sustainability and scale-up under real world conditions was strengthened by the utilization of existing health system capacities and partnerships, such as health worker facilitators, local and multi-sector recruitment channels, and community-based primary care [85].

This study highlights several lessons and challenges regarding study and intervention feasibility that require consideration. One such issue was the screening procedures used to identify caregivers at risk of perpetrating violence against children, in which only one out of 63 participants did not meet the cut-off criteria. While this may suggest that the threshold was too low, it may also have been due to Village Health Volunteers and teachers seeking out higher risk families during the identification and referral process. Nonetheless, the screening interview should either be eliminated, or the threshold increased to target caregivers reporting higher rates or more severe forms of maltreatment.

Further, recruitment procedures should also be carefully revisited. First, the relatively moderate consent rate and rationales given for declining to participate suggest that many working caregivers were not available to attend the program, even on Sundays. Second, the careful presentation of the program to potential participants was important. During recruitment, a Village Health Volunteer in one area pointed out a caregiver to research staff as abusive and “needing to participate in the program.” This led to clarification by study researchers that the public shaming of caregivers during the referral process may contribute to stigmatization, as well as increase the likelihood that caregivers would decline to participate due to perceptions that the program would be punitive rather than supportive. Third, teachers in one sub-district were important sources of caregiver referrals, suggesting that Early Child Development Centers and primary schools should also be included in future recruitment efforts. Fourth, the use of both phone calls and home visits accompanied by familiar Village Health Volunteers were necessary in many cases, as caregivers contacted via “cold calling” alone were more suspicious of study researchers or simply did not answer calls. Finally, only four male referrals were provided, with only one consenting to participate. Low rates of male engagement in parenting programs are a common challenge across countries [86–88]; in Thailand, as in South Africa, this may reflect the social norm that childrearing is a role primarily reserved for women [89, 90]. Targeted outreach to male primary caregivers should thus be incorporated into RCT study procedures, while research on barriers to male enrolment and attendance in Thailand should be conducted [91].

The study also underscored the necessity of combining the program with well-articulated child protection and adult welfare referral procedures, as well as the importance of familiarity with local laws and regulations and close collaboration with local service providers. During the course of data collection and program delivery, 14 adults and five children were referred for child protection, social welfare services, or clinical assessments, with most referrals requested by caregivers themselves.

The study also provided a valuable opportunity to assess outcome measurement reliability and suitability. This was of particular relevance given that most of the outcome measurements had not been validated in Thailand. Examinations of Cronbach’s alpha, showing that only 59.3% of values fell within acceptable ranges (see Additional file 2) [83], may reflect weak internal consistency of scales. However, Cronbach’s alpha itself does not provide sufficient evidence of instrument quality, and is less informative for scales that measure several constructs at once [92]. Despite such considerations, the questionable reliability of these instruments suggests that metric invariance should be assessed prior to further use. Furthermore, the pilot tested the suitability of the HOME Inventory as a mixed interview and observational assessment tool. It was substantially adapted and shortened from the original, allowing for application to the range of child ages in the sample and administration within 30–60 min. Although the use of videotaped observations to establish inter-rater reliability between independent assessors would strengthen instrument validity, the study findings suggest that the instrument can sensitively detect observed and reported changes in maltreatment rates and caregiver-child relationships.

Study findings also support the feasibility of delivering an adapted version of PLH-YC for low-income families – including grandparents – in Udon Thani. High rates of program enrolment, attendance, and completion, with no dropouts, suggest that the intervention is relevant and acceptable to participants across a wide age range [93]. Given that one in five children in Thailand reside in households without either biological parent, with many of their primary caregivers being grandparents, it is important that the program is able to meet the caregiving needs of this older population [94]. However, several aspects should be considered prior to future delivery. The provision of travel compensation for all parenting group participants, as well as transport for two groups, may have contributed to high program attendance. This travel compensation in the amount of 150 Baht (4.95 USD), was nearly half of the daily minimum wage (315 Baht or 10.40 USD) for Udon Thani province [95]. Removal of these benefits may result in lower rates of engagement [96]. Moreover, given the large proportion of older participants with visual and mobility impairments, as well as low levels of education and literacy, program materials (e.g., illustrated story posters, parent handbook), the use of mobile text messaging, and disability-inclusive transport need further review to reduce potential obstacles to accessibility.

Following participation in PLH-YC, caregivers reported reductions on all primary outcomes of interest, with moderate size effects on overall child maltreatment and physical abuse, and small effects on emotional abuse. The HOME Inventory instrument also demonstrated moderate size effects on abusive and harsh parenting. Analyses of secondary outcomes showed promising results across almost all outcome measures (22 out of 24). Caregivers reported decreases in dysfunctional parenting; poor child monitoring and supervision; neglect; depression, anxiety, and stress; attitudes supporting physical punishment and harsh discipline; daily and past month child behavior problems; parent sense of inefficacy; and intimate partner violence. There were also improvements in caregiver reports of positive parenting, daily parenting behavior, as well as combination interview and observational assessments of caregiver-child relationships. Qualitative findings converged with quantitative results, with perceived key mechanisms of change including strengthened caregiver-child relationships; increase child compliance and reduced child behavior problems; improved attitudes and strategies toward discipline; as well as improved management of parental stress. There was no evidence of adverse effects.

Although the study lacked a control group and was not designed to test causal impact, findings suggest that this 8-session version of PLH-YC could lead to an array of positive effects, despite a 33% reduction in dosage from the original 12-session version, and is worth testing more rigorously in a subsequent RCT. The program’s overall hypothesized theory of change thus appears viable and complements the mechanisms of change perceived by caregivers and facilitators. However, the study raises two questions. One is regarding the lesser impact on reported rates of child emotional abuse in comparison to child physical abuse, along with qualitative findings indicating that some caregivers may still be utilizing or switching to such practices. This suggests that program content may need to specifically address this form of violence, and that ICAST-TC measures should be expanded to encompass a broader range of emotional abuse items (e.g., telling the child “I do not or will not love you”). Another question concerns the effects of the intervention on intimate partner relationships, as domestic violence was significantly reduced yet coercion and negotiation did not significantly change. This requires empirical examinations of potential moderators of impact and possible diffusion effects.

This study has a number of additional limitations. Foremost, findings from a pre-post, non-randomized study cannot reliably be attributed to the intervention, and can only be indicative of potential program results [97]. Second, we used a relatively limited range of data sources. Due to their young age, we did not utilize child reports or interview children, which could have corroborated findings from caregivers, observers, and facilitators. The inclusion of other reports and individual interviews, such as with other family members, as well as caregiver report and observational assessments with other children in the household, would have provided yet further data on potential impacts outside the dyadic caregiver-target child relationship. While we also included observational measures and periodic daily reports, our reliance mainly on self-report measures and a limited set of individual caregiver interviews therefore raises concerns of recall bias and the influence of social desirability. A third limitation is that post-test assessments and individual interviews were conducted only 1 month after intervention; thus, it is uncertain whether effects were sustained, diminished, or delayed over the medium and long term [98]. Fourth, while internal consistency is only one aspect of instrument validity, the low Cronbach’s alpha scores for many of the measures suggest cautious interpretation of study findings. Fifth, a large number of outcomes were analyzed, which raises the risks of false positive effects. However, no statistical adjustments for multiple tests were undertaken due to the potential for overcorrection and obscuring of treatment effects [99]. Nonetheless, checking with a conservative Bonferroni correction (α = 0.05/32), including different time point comparisons using the PDR, 23 out of 28 effects remained statistically significant, except for proactive parenting, poor child monitoring and supervision, parental anxiety and stress, and IPV. Finally, given the time constraints for this study, we did not conduct qualitative interviews with caregivers who did and did not do well following program completion based on outcome analyses. This would have yielded deeper insights on underlying mechanisms of change and allowed us to better isolate the parenting techniques perceived as having the most and least utility.

Despite such limitations, the study has many strengths. First, the strong involvement of high-level public health officials and local practitioners raises prospects for the scaling up of the intervention – should positive effects be sustained in subsequent RCT testing. Second, the indication by caregivers through individual interviews that they shared acquired program skills with household members may engender diffusion effects and public demand that fosters scalability. A third strength was the inclusion of observer ratings, regarded as a “gold standard” in objective evaluations of changes in caregiver-child interactions [100], as well as periodic daily reports, which avoid the demands of aggregate recall over multiple days or for estimates of behavior frequency [101]. Fourth, significant reductions in child maltreatment rates converged with qualitative data. This demonstrates the value of adopting mixed methods approaches to feasibility pilots, as it allows for the maximizing of strengths from each type of data. Finally, the inclusion of audio-CASI to administer more sensitive items regarding child maltreatment and IPV assisted in encouraging disclosures and reducing risks of social desirability bias.

## Conclusions

This mixed methods study is the first known evaluation of an evidence- and group-based parenting intervention delivered within the public health system in Thailand, with several notable findings. First, it shows the feasibility of study evaluation approaches, including data collection methods and the absence of adverse effects, although the reliability of many outcome measurements is uncertain. Second, it also demonstrates program feasibility and the preliminary effectiveness of a parenting intervention in reducing child physical and emotional violence and other related outcomes within low-income families in Thailand, with perceived key mechanisms of change corroborating some of these effects. Finally, it highlights that close collaboration with policymakers and local practitioners is possible, thus improving prospects for program scalability and sustainability through the utilization of existing routine public health service staff and delivery systems. While further research is necessary to determine program effectiveness before such expansion efforts should be undertaken, these initial findings suggest a promising contribution toward violence prevention in Thailand and other LMICs more broadly.

## Supplementary Information

**Additional file 1.** Overview of Parenting for Lifelong Health – Young Children adapted session content for Thailand. Table comparing original 12-session version content with the adapted 8-session version.

**Additional file 2.** Reliability of outcome instruments using pooled imputed datasets. Table with Cronbach’s alpha values for each outcome instrument.

**Additional file 3.** Complete case analyses. Outcomes at pre- and post-test for complete cases, and PDR assessment outcomes and comparisons at four time points for complete cases. Two tables with outcome data using complete case analysis.

## Data Availability

The datasets used and/or analysed during the current study available from the corresponding author on reasonable request.

## Abbreviations

APQ: Alabama Parenting Questionnaire
CTS2S: Revised Conflict Tactics Scale Short Form
DASS-21: Depression, Anxiety, and Stress Scale Short Form
ECBI: Eyberg Child Behavior Inventory
HOME: Home Observation for Measurement of the Environment
ICAST: International Society for the Prevention for Child Abuse and Neglect (ISPCAN) Child Abuse Screening Tools
IPV: Intimate partner violence
LMIC: Low- and middle-income country
MICS: Multiple Indicator Cluster Survey
MOPH: Ministry of Public Health
MORU: Mahidol-Oxford Tropical Medicine Research Unit
NESDB: National Economic and Social Development Board
NSO: National Statistical Office
PARYC: Parenting Young Children Scale
PDR: Parent Daily Report
PLH-YC: Parenting for Lifelong Health for Young Children
PS: Parenting Scale
RCT: Randomized controlled trial
UN: United Nations
UNFPA: United Nations Population Fund
UNICEF: United Nations Children’s Fund
VAC: Violence against children
WHO: World Health Organization

## Footnotes

1 Manuscript in development.

2 Manuscript in submission.

3 Manuscript in development.
