# Supplementary material for: Feasibility pilot of an adapted parenting program embedded within the Thai public health system
Source: BMC Public Health. 2021 May 29;21:1009. doi: 10.1186/s12889-021-11081-4 (PMC8164235; doi:10.1186/s12889-021-11081-4)
Supplement: Supplementary file 1 — Additional file 1. Overview of Parenting for Lifelong Health – Young Children adapted session content for Thailand. Table comparing original 12-session version content with the adapted 8-session version. [file 12889_2021_11081_MOESM1_ESM.docx]

# Feasibility pilot of an adapted parenting program embedded within the Thai public health system

Authors: Amalee McCoy,^ab^ Jamie M. Lachman,^ac^ Catherine L. Ward,^d^ Sombat Tapanya,^b^ Tassawan Poomchaichote,^b^ Jane Kelly,^e^ Mavuto Mukaka,^bf^ Phaik Yeong Cheah,^bf^ and Frances Gardner^a^

Corresponding author: Amalee McCoy, Centre for Evidence-Based Intervention, Department of Social Policy and Intervention, University of Oxford, Barnett House, 32 Wellington Square, Oxford OX1 2ER, United Kingdom; Email: amalee.mccoy@gmail.com

^a^Centre for Evidence-Based Intervention, Department of Social Policy and Intervention, University of Oxford, Oxford, United Kingdom; ^b^Mahidol Oxford Tropical Medicine Research Unit, Faculty of Tropical Medicine, Mahidol University, Bangkok, Thailand; ^c^MRC/CSO Social and Public Health Sciences Unit, University of Glasgow; ^d^Department of Psychology, University of Cape Town, Cape Town, South Africa; ^e^Centre for Social Science Research, University of Cape Town, Cape Town, South Africa; ^f^Nuffield Department of Clinical Medicine, University of Oxford, Oxford, United Kingdom.

#### Additional File 1. Overview of Parenting for Lifelong Health – Young Children adapted session content for Thailand

| **Session** | **Original 12-session version** | **Session** | **Adapted 8-session version** |
| --- | --- | --- | --- |
| **1** | Program introduction and spending one-on-one time with your child | **1** | Program introduction and spending one-on-one time with your child (inclusion of some content on child-directed speech) |
| **2** | Child-directed speech during one-on-one time |  |  |
| **3** | Talking about feelings | **2** | Talking about feelings |
| **4** | Praising and rewarding | **3** | Praising and rewarding |
| **5** | Instruction-giving | **4** | Instruction-giving |
| **6** | Household rules and daily routines | **5** | Household rules and daily routines |
| **7** | Redirecting negative behaviors | **6** | Ignoring negative attention seeking and demanding behaviors (inclusion of some content on ignoring) |
| **8** | Ignoring negative attention seeking and demanding behaviors |  |  |
| **9** | Using consequences to support compliance | **7** | Using consequences to support compliance |
| **10** | Time-out for aggressive and destructive behaviors | **8** | Problem solving, talking about difficult issues, and reflecting and moving on, followed by community celebration (exclusion of time-out content) |
| **11** | Problem solving and talking about difficult issues |  |  |
| **12** | Reflecting and moving on, followed by community celebration |  |  |
