# Supplementary material for: Feasibility pilot of an adapted parenting program embedded within the Thai public health system
Source: BMC Public Health. 2021 May 29;21:1009. doi: 10.1186/s12889-021-11081-4 (PMC8164235; doi:10.1186/s12889-021-11081-4)
Supplement: Supplementary file 2 — Additional file 2. Reliability of outcome instruments using pooled imputed datasets. Table with Cronbach’s alpha values for each outcome instrument. [file 12889_2021_11081_MOESM2_ESM.docx]

# Feasibility pilot of an adapted parenting program embedded within the Thai public health system

Authors: Amalee McCoy,^ab^ Jamie M. Lachman,^ac^ Catherine L. Ward,^d^ Sombat Tapanya,^b^ Tassawan Poomchaichote,^b^ Jane Kelly,^e^ Mavuto Mukaka,^bf^ Phaik Yeong Cheah,^bf^ and Frances Gardner^a^

Corresponding author: Amalee McCoy, Centre for Evidence-Based Intervention, Department of Social Policy and Intervention, University of Oxford, Barnett House, 32 Wellington Square, Oxford OX1 2ER, United Kingdom; Email: amalee.mccoy@gmail.com

^a^Centre for Evidence-Based Intervention, Department of Social Policy and Intervention, University of Oxford, Oxford, United Kingdom; ^b^Mahidol Oxford Tropical Medicine Research Unit, Faculty of Tropical Medicine, Mahidol University, Bangkok, Thailand; ^c^MRC/CSO Social and Public Health Sciences Unit, University of Glasgow; ^d^Department of Psychology, University of Cape Town, Cape Town, South Africa; ^e^Centre for Social Science Research, University of Cape Town, Cape Town, South Africa; ^f^Nuffield Department of Clinical Medicine, University of Oxford, Oxford, United Kingdom.

#### Additional File 2. Reliability of outcome instruments using pooled imputed datasets

| **Instrument** | **Cronbach’s alpha** | | **Number of items** |
| --- | --- | --- | --- |
|  | **Pre-test** | **Post-test** |  |
| ICAST-T Overall | **0.73** | 0.67 | 25 |
| Physical abuse | 0.54 | 0.63 | 15 |
| Emotional abuse | **0.70** | 0.64 | 10 |
| Neglect subscale | 0.45 | 0.41 | 6 |
| Attitudes toward harsh discipline | - 0.47 | 0.26 | 4 |
| Inefficacy | 0.64 | 0.67 | 2 |
| HOME Inventory Abusive & harsh parenting | 0.50 | 0.68 | 6 |
| HOME Inventory Overall parent-child relationships | **0.72** | **0.70** | 27 |
| Parental responsivity | **0.73** | 0.68 | 16 |
| Encouragement of child maturity | 0.67 | 0.65 | 6 |
| PARYC Overall positive parenting | **0.88** | **0.91** | 21 |
| Supporting positive behavior | 0.69 | **0.83** | 7 |
| Setting limits | **0.86** | **0.80** | 7 |
| Proactive parenting | **0.73** | **0.83** | 7 |
| APS Dysfunctional parenting | **0.70** | **0.74** | 10 |
| APQ Poor child monitoring & supervision | **0.73** | **0.76** | 11 |
| DASS Overall | **0.92** | **0.89** | 21 |
| Depression | **0.85** | **0.78** | 7 |
| Anxiety | **0.78** | 0.69 | 7 |
| Stress | **0.76** | **0.76** | 7 |
| ECBI Intensity | **0.91** | **0.93** | 36 |
| ECBI Problems | **0.96** | **0.96** | 36 |
| CTS2S Intimate partner violence | 0.14 | 0.05 | 6 |
| Negotiation | 0.15 | **0.76** | 2 |
| WHO Intimate partner coercion | 0.62 | 0.54 | 10 |
| PDR Child problem behavior | **0.87** | **0.82** | 34 |
| PDR Positive parenting behavior | 0.37 | 0.43 | 9 |

Acceptable levels of reliability (0.70 to >0.90) are in bold
