# Supplementary material for: Feasibility pilot of an adapted parenting program embedded within the Thai public health system
Source: BMC Public Health. 2021 May 29;21:1009. doi: 10.1186/s12889-021-11081-4 (PMC8164235; doi:10.1186/s12889-021-11081-4)
Supplement: Supplementary file 3 — Additional file 3. Complete case analyses. Outcomes at pre- and post-test for complete cases, and PDR assessment outcomes and comparisons at four time points for complete cases. Two tables with outcome data using complete case analysis. [file 12889_2021_11081_MOESM3_ESM.docx]

**Feasibility pilot of an adapted parenting program embedded within the Thai public health system**

Authors: Amalee McCoy,^ab^ Jamie M. Lachman,^ac^ Catherine L. Ward,^d^ Sombat Tapanya,^b^ Tassawan Poomchaichote,^b^ Jane Kelly,^e^ Mavuto Mukaka,^bf^ Phaik Yeong Cheah,^bf^ and Frances Gardner^a^

Corresponding author: Amalee McCoy, Centre for Evidence-Based Intervention, Department of Social Policy and Intervention, University of Oxford, Barnett House, 32 Wellington Square, Oxford OX1 2ER, United Kingdom; Email: [amalee.mccoy@gmail.com](mailto:amalee.mccoy@gmail.com)

^a^Centre for Evidence-Based Intervention, Department of Social Policy and Intervention, University of Oxford, Oxford, United Kingdom; ^b^Mahidol Oxford Tropical Medicine Research Unit, Faculty of Tropical Medicine, Mahidol University, Bangkok, Thailand; ^c^MRC/CSO Social and Public Health Sciences Unit, University of Glasgow; ^d^Department of Psychology, University of Cape Town, Cape Town, South Africa; ^e^Centre for Social Science Research, University of Cape Town, Cape Town, South Africa; ^f^Nuffield Department of Clinical Medicine, University of Oxford, Oxford, United Kingdom.

#### Additional File 3.

#### Outcomes at pre- and post-test for complete cases

| **Outcome** | **Pre-test**  ***M* (*SD*)** | **Post-test**  **M (*SD*)** | **Test statistic* (*p*)** | **Effect size^b^** | ***N***^c^ |
| --- | --- | --- | --- | --- | --- |
| **Primary outcome** | | | | | |
| Child maltreatment - physical & emotional abuse (ICAST-T), /200^a^ | 7.86 (7.07) | 3.67 (3.82) | **- 4.96 (<0.001)** | **- 0.59** | 58 |
| Physical abuse subscale, /120 | 3.64 (3.97) | 1.33 (2.02) | **- 4.49 (<0.001)** | **- 0.58** | 58 |
| Emotional abuse subscale, /80 | 4.22 (4.45) | 2.35 (2.92) | **- 4.22 (<0.001)** | **- 0.42** | 58 |
| HOME Inventory: Abusive & harsh parenting, /6 | 1.12 (1.07) | 0.51 (0.74) | **- 3.99 (<0.001)** | **- 0.57** | 57 |
| **Secondary outcomes** | | | | | |
| Overall positive parenting (PARYC), /126 | 68.78 (17.20) | 80.81 (16.40) | **5.06^d^ (<0.001)** | **0.70** | 59 |
| Supporting positive behavior subscale, /42 | 24.78 (5.64) | 28.75 (5.18) | **5.02^d^ (<0.001)** | **0.70** | 59 |
| Setting limits subscale, /42 | 21.81 (7.93) | 27.0 (6.17) | **5.00^d^ (<0.001)** | **0.65** | 59 |
| Proactive parenting subscale, /42 | 22.19 (6.96) | 25.07 (7.35) | **2.66^d^ (0.010)** | **0.41** | 59 |
| Dysfunctional parenting (PS), /70 | 26.29 (9.05) | 19.92 (8.20) | **- 5.16 (<0.001)** | **- 0.70** | 59 |
| Poor child monitoring & supervision (APQ), /55 | 15.93 (4.95) | 14.46 (4.17) | **- 2.71 (0.007)** | **- 0.30** | 59 |
| Neglect (ICAST-T), /48 | 1.33 (2.26) | 0.328 (0.76) | **- 3.35 (0.001)** | **- 0.44** | 58 |
| Overall depression, anxiety, and stress (DASS-21), /126 | 7.88 (7.25) | 4.32 (4.85) | **- 3.46 (0.001)** | **- 0.49** | 59 |
| Depression subscale, /42 | 4.78 (5.81) | 2.20 (3.56) | **- 3.14 (0.001)** | **- 0.44** | 59 |
| Anxiety subscale, /42 | 4.710.51 (4.95) | 2.48 (3.16) | **- 3.10 (0.002)** | **- 0.45** | 59 |
| Stress subscale, /42 | 6.27 (5.04) | 3.97 (4.36) | **- 2.79 (0.005)** | **- 0.46** | 59 |
| Attitudes supporting physical punishment (MICS), /23 | 2.02 (1.21) | 0.93 (1.03) | **- 4.16 (<0.001)** | **- 0.90** | 59 |
| Attitudes toward harsh discipline (ICAST-T), /20 | 10.27 (2.02) | 8.46 (1.74) | **- 5.05 (<0.001)** | **- 0.90** | 59 |
| Child behavior problems (ECBI) – Intensity subscale, /252 | 101.41 (28.71) | 78.24 (27.61) | **- 5.31 (<0.001)** | **- 0.81** | 59 |
| Child behavior problems (ECBI) – Problems subscale, /36 | 7.64 (9.41) | 1.80 (4.86) | **- 5.20 (<0.001)** | **- 0.62** | 59 |
| Parent sense of inefficacy subscale (ICAST-T), /16 | 3.78 (3.45) | 1.90 (2.43) | **- 3.90 (<0.001)** | **- 0.54** | 58 |
| HOME Inventory: Overall caregiver-child relationships, /27 | 21.11 (3.66) | 24.07 (2.73) | **5.23 (<0.001)** | **0.81** | 57 |
| Parental responsivity subscale, /16 | 13.18 (2.59) | 14.30 (1.95) | **3.43 (0.001)** | **0.43** | 57 |
| Encouragement of child maturity subscale, /6 | 4.46 (1.44) | 5.56 (0.93) | **4.80 (<0.001)** | **0.77** | 57 |
| Intimate partner violence subscale (CTS2S)/48 | 1.05 (1.59) | 0.548 (1.11) | **- 2.202 (0.028)** | **- 0.31** | 42 |
| Intimate partner negotiation subscale (CTS2S)/16 | 3.05 (3.00) | 3.36 (4.37) | - 0.24 (0.811) |  | 42 |
| Intimate partner coercion (WHO), /80 | 3.12 (4.65) | 2.19 (3.68) | - 1.41 (0.158) |  | 42 |

Statistically significant differences (*p* < 0.05) between pre- and post-test are in bold

*Unless otherwise noted, standardized test statistics are from Wilcoxon Signed Rank tests

^a^Value indicates the maximum possible total score

^b^Cohen’s *d*

^c^*N* for complete cases varies based on the scale, ranging from 42 to 59 caregivers

^d^Test statistic from paired samples t-test

#### PDR assessment outcomes and comparisons at four time points for complete cases

| **Outcome** | **Pre-test**  ***M* (*SD*)** | **PDR#2 *M* (SD)** | **PDR #3 *M* (*SD*)** | **Post-test *M* (*SD*)** | **Test statistic* (*p*)** | ***N*** | **Comparison** | **Test statistic* (*p*)** | ***SE*** | **Effect size^b^** |
| --- | --- | --- | --- | --- | --- | --- | --- | --- | --- | --- |
| Parent daily report (PDR) on child problem behavior, /34^a^ | 6.93 (5.59) | 6.81 (5.23) | 4.78 (4.44) | 3.73 (3.89) | **35.89 (<0.001)** | 59 | Pre-test, PDR#2 | - 0.07 (0.775) | 0.24 |  |
|  |  |  |  |  |  |  | PDR#2, PDR#3 | **- 0.86 (<0.001)** | 0.24 | **- 0.39** |
|  |  |  |  |  |  |  | PDR#3, Post-test | - 0.24 (0.318) | 0.24 |  |
|  |  |  |  |  |  |  | Pre-test, Post-test | **- 1.03 (<0.001)** | 0.24 | **- 0.57** |
| Parent daily report (PDR) on positive parenting behavior, /9 | 7.15 (1.42) | 7.95 (1.09) | 8.25 (0.98) | 8.31 (1.00) | **44.02 (<0.001)** | 59 | Pre-test, PDR#2 | **0.74 (0.002)** | 0.24 | **0.56** |
|  |  |  |  |  |  |  | PDR#2, PDR#3 | 0.37 (0.117) | 0.24 |  |
|  |  |  |  |  |  |  | PDR#3, Post-test | 0.06 (0.803) | 0.24 |  |
|  |  |  |  |  |  |  | Pre-test, Post-test | **1.17 (<0.001)** | 0.24 | **0.81** |

Statistically significant differences (*p* < 0.05) between comparisons are in bold

*Test statistics are from Friedman’s ANOVA

^a^Value indicates the maximum possible total score

^b^Cohen’s *d*
